# 

**Published:** 1952-10

**Authors:** 


THE BRISTOL

MEDICO-CHIRURGICAL JOURNAL

*A Journal of the Medical Sciences for the*

WEST OF ENGLAND AND SOUTH WALES

VOL. 69 OCTOBER 1952 NO. 252

### Contents

*PUBLISHED QUARTERLY*

*ANNUAL SUBSCRIPTION 16/- POST FREE*

PUBLISHED UNDER THE AUSPICES OF THE
BRISTOL MEDICO-CHIRURGICAL SOCIETY

### Editorial Committee

MR. H. KEITH LUCAS, 60 St. John's Road, Bristol 8. *Editor.*

DR. J. E. CATES, Dept. of Medicine, Bristol Royal Infirmary } *Assistant Editors.*
DR. A. H. GALE, The University, Bristol 8 }

DR. G. E. F. SUTTON, 1 Pembroke Road, Bristol 8. *Treasurer.*

DR. A. M. MACLACHLAN, 167 Redland Road, Bristol 6.

DR. F. J. Y. WOOD, Dept. of Medicine, Bristol Royal Infirmary.

*Local Correspondents*

*Bath* . . Mr. A. D. BATEMAN, 3 The Circus, Bath.
*Exeter* . . Dr. L. N. JACKSON, Mount Jocelyn, Crediton, Devon.
*Gloucester* . Dr. R. F. JARRETT, 9 College Green, Gloucester.
*Newport* . Mr. J. L. D. WILLIAMS, The Knoll, 145 Stow Hill, Newport, Mon.
*Plymouth* . Dr. C. R. CROFT, Lexden, Hartley Av., Plymouth.
*Taunton* . Dr. M. MCALLEY, 1 The Crescent, Taunton.
*Torquay* . Dr. A. ROBINSON THOMAS, 20 Devon Square, Newton Abbot, Devon.
*Truro* . . Dr. F. D. M. HOCKING, St. Andrews, Perranporth, Cornwall.
*Yeovil* . . Mr. J. A. VERE NICHOLL, Knapp House, Yeovil, Somerset.

### NOTICE TO CONTRIBUTORS

Original articles are invited, provided they have not been published elsewhere. Notes of forthcoming events, meetings held, degrees conferred, staff appointments, and other local news are welcomed. The editorial committee reserves the right to make literary corrections.

Illustrations should always be supplied ready for reproduction. All re-touchings or other operations required will be charged to the author. Line drawings should be drawn in Indian ink. We regret that we must ask authors to pay for making blocks, if this is necessary.

J. W. ARROWSMITH LTD., SMALL STREET,
BRISTOL.

### *Appointments*

| *Bristol* | *Appointment* | *Formerly* |
|---|---|---|
| ADAMS, AILEEN K., M.B. | Senior Registrar in Anaesthetics, Frenchay Hospital. | Registrar in Anaesthetics, Frenchay Hospital. |
| BAILY, R. A. J., M.B., M.R.C.S. | Registrar in Orthopaedics, United Bristol Hospitals. | Senior Orthopaedic Registrar, Southampton. |
| BROCKWAY, E. N., M.B., F.R.C.S. | Senior Registrar in Orthopaedics, United Bristol Hospitals. | Registrar in Orthopaedics, United Bristol Hospitals. |
| CHITTY, K., M.B. | Senior Registrar in Genito-Urinary Department, United Bristol and Southmead Hospitals. | Surgical Registrar, St. Pancras Hospital. |
| COSH, J. A., M.D., M.R.C.P. | Assistant Physician, United Bristol Hospitals. | |
| CROSS, E. G. W., M.R.C.S., D.P.M., | Senior Registrar in Psychiatry, Bristol Mental Hospitals. | Registrar, Bristol Mental Hospitals. |
| DALGLEISH, P. G., M.D., M.R.C.P. | Lecturer in Medicine, University of Bristol. | Registrar in Medicine, United Bristol Hospitals. |
| FREEMAN, J., M.B., F.R.C.S. | Senior Registrar (Ear, Nose and Throat), United Bristol Hospitals. | Registrar, E.N.T., United Bristol Hospitals. |
| GLANVILLE, J. D., M.B. | Registrar (Ear, Nose and Throat), United Bristol Hospitals. | Registrar, E.N.T., St. James' Hospital. |
| HOLLIS, P. W. T., M.B. | Assistant Medical Director, Mass Radiography Unit. | Registrar, Thoracic Surgery, Devon & Cornwall. |

| | | |
|---|---|---|
| KIRKPATRICK, C. R., M.R.C.S. | Senior Registrar in Anaesthetics, United Bristol Hospitals. | Senior Registrar in Anaesthetics, Dundee Royal Infirmary. |
| LEVERTON, J. C. S., M.B., M.D., M.R.C.S., M.R.C.O.G. | Registrar in Obstetrics and Gynaecology, United Bristol Hospitals. | Travelling Scholarship, U.S.A. |
| MACGREGOR, A. R., L.D.S., R.C.S. | Registrar in Dental Surgery, Bristol Dental Hospital. | Tutor in Dental Surgery, Bristol. |
| MACGREGOR, W. G., M.B., M.R.C.O.G. | Senior Registrar in Obstetrics and Gynaecology, Southmead Hospital. | Registrar, Department of Obstetrics and Gynaecology, Oxford United Hospitals. |
| MCGWINN, P. L., M.B., D.A. | Registrar in Anaesthetics, Frenchay Hospital. | Locum Anaesthetic Registrar, Frenchay. |
| MICHAELS, L., M.B. | Demonstrator in Bacteriology, University of Bristol. | Senior House Officer, United Bristol Hospitals. |
| MORGAN, O. T. L., M.B. | Registrar in Anaesthetics, United Bristol Hospitals. | Senior Resident Assistant Anaesthetist, Middlesex Hospital. |
| NEVE, C. R., M.B., F.R.C.S. | Senior Registrar in General Surgery, Southmead Hospital. | Registrar in General Surgery, United Bristol Hospitals. |
| RUEBNER, B., M.B. | Demonstrator in Pathology, University of Bristol. | Senior House Officer, United Bristol Hospitals |
| WATSON, P. C., M.R.C.S. | Senior Registrar in General Surgery, United Bristol Hospitals. | Senior Registrar in General Surgery, Southmead Hospital. |
| ALLCOCK, J. M., M.B., F.R.C.S. | Registrar in Radiology, Bristol Clinical Area (part-time). | Southern Rhodesia Medical Service. |
| MOORE, E. W., M.B. | Registrar in Radiology, Bristol Clinical Area (part-time). | House Physician to Senior Consultant for Diseases of Chest, Slade Hospital, Oxford. |

| *Bath* | *Appointment* | *Formerly* |
|---|---|---|
| BAZELEY, R. W., M.B., M.R.C.S. | Clinical Assistant in General Medicine, Bath. | Registrar in Medicine, Prince of Wales General Hospital, Tottenham. |
| BOLTON, J. R., M.D., M.R.C.P. | Consultant in General Medicine (Geriatrics), Bath. | Assistant Director (late 1st Asst.), Med. Unit. Lond. Hosp. |
| BURROWES, W. L., M.B., M.R.C.P. | Clinical Assistant in General Medicine, Bath. | Clinical Assistant in General Medicine, Bath. |
| DE FONSEKA, C. P., M.B. | Registrar in General Surgery, Bath. | Senior Resident Officer, St. Bart's, Rochester. |
| LINDSAY, I., M.B., F.R.C.S. | Registrar in General Surgery, Bath. | Junior Registrar, S. Devon and E. Cornwall Hospital. |

| | | |
|---|---|---|
| McSwiney, M. J., M.B. | Assistant Anaesthetist, Bath. | Locum Anaesthetist, Bath Clinical Area. |
| Miles, E. W., M.B., M.R.C.S., D.C.H. | Clinical Assistant in Paediatrics, Bath. | General Practice, Bath. |
| Poyner-Wall, Phyllis, M.B., M.R.C.S. | Registrar in Diseases of Chest, Winsley Chest Hospital, Nr. Bath. | Senior House Officer, Winsley Chest Hospital, Nr. Bath. |
| Richardson, R. E., M.R.C.S., L.R.C.P. | Registrar in General Medicine, Bath. | S.H.O., St. Martin's Hospital. |
| *South Somerset* | | |
| Manning, Helen, M.B. | Clinical Assistant in E.N.T. Surgery, South Somerset. | Part-time work at Taunton and Somerset Hospital. |
| Russell, J. G., M.B., M.R.C.P., D.P.M. | Consultant Psychiatrist, South Somerset. | Senior Registrar St. Ebba's, Epsom. |
| *Gloucestershire* | | |
| Nevin, H. M., M.B., D.P.H. | Assistant Pathologist, North Gloucestershire. | Assistant Bacteriologist, Colindale. |
| *Exeter* | | |
| Edgcumbe, J. O. P., M.B., M.R.C.S. | Consultant Pathologist, Exeter. | Assistant Clinical Pathologist, National Hospital, Queen Square. |
| Warren, C. P., M.R.C.S., M.B. | Consultant Pathologist, Exeter (Torquay). | Consultant Pathologist, Exeter and Mid-Devon Clinical Area. |
| *Cornwall and Plymouth* | | |
| Bourns, H. K., M.B., F.R.C.S. | Senior Registrar in General Surgery, Plymouth (Transfer). | Senior Registrar in General Surgery, United Bristol Hospitals. |
| Bussell, L. J., D.M., M.R.C.P. | Senior Registrar in General Medicine, South Devon and East Cornwall. | Senior Registrar, Royal Infirmary, Sheffield. |
| Dumoulin, J. G., M.D., M.R.C.S., M.R.C.O.G. | Consultant in Obstetrics and Gynaecology, Plymouth. | 1st Asst. Obst. Unit, Univ. Coll. Hosp. |
| Harrison, G. E., M.B. | Assistant Physician in Chest Diseases, West Cornwall (Tehidy). | Physician Superintendent, Nab Top Sanatorium, Marble, Cheshire (S.H.M.O. grade). |
| Noble, N., M.R.C.S., M.R.C.O.G., F.R.C.S.E. | Senior Registrar in Obstetrics and Gynaecology, South Devon and East Cornwall Hospital. | Senior Registrar, Camborne-Redruth Miners' and General Hospital. |
| Prus, N., Med. Dip., M.D. (Lwow). | Assistant Psychiatrist, West Cornwall. | Junior Hospital Medical Officer, Bristol Mental Hospitals. |

| | | |
|---|---|---|
| RAY, R. L., M.B., M.R.C.S. | Assistant Physician in Chest Diseases, West Cornwall. | Registrar, Harefield Hospital. |
| *Weston-super-Mare* | | |
| TEMPLE, J. LL., M.B., F.R.C.S. | Consultant Surgeon, Weston-super-Mare. | Senior Surgical Registrar, St. George's Hospital, S.W.1. |

